# Monitoring Mixing Processes Using Ultrasonic Sensors and Machine Learning

**DOI:** 10.3390/s20071813

**Published:** 2020-03-25

**Authors:** Alexander L. Bowler, Serafim Bakalis, Nicholas J. Watson

**Affiliations:** Faculty of Engineering, University of Nottingham, University Park, Nottingham, NG7 2RD, UK; enxab17@exmail.nottingham.ac.uk (A.L.B.); ezzsb3@exmail.nottingham.ac.uk (S.B.)

**Keywords:** food and drink manufacturing, industry 4.0, digital manufacturing, mixing, ultrasonic sensors, machine learning, convolutional neural networks, long short-term memory neural networks, wavelet transform

## Abstract

Mixing is one of the most common processes across food, chemical, and pharmaceutical manufacturing. Real-time, in-line sensors are required for monitoring, and subsequently optimising, essential processes such as mixing. Ultrasonic sensors are low-cost, real-time, in-line, and applicable to characterise opaque systems. In this study, a non-invasive, reflection-mode ultrasonic measurement technique was used to monitor two model mixing systems. The two systems studied were honey-water blending and flour-water batter mixing. Classification machine learning models were developed to predict if materials were mixed or not mixed. Regression machine learning models were developed to predict the time remaining until mixing completion. Artificial neural networks, support vector machines, long short-term memory neural networks, and convolutional neural networks were tested, along with different methods for engineering features from ultrasonic waveforms in both the time and frequency domain. Comparisons between using a single sensor and performing multisensor data fusion between two sensors were made. Classification accuracies of up to 96.3% for honey-water blending and 92.5% for flour-water batter mixing were achieved, along with R^2^ values for the regression models of up to 0.977 for honey-water blending and 0.968 for flour-water batter mixing. Each prediction task produced optimal performance with different algorithms and feature engineering methods, vindicating the extensive comparison between different machine learning approaches.

## 1. Introduction

The world is experiencing the fourth industrial revolution where digital technologies such as artificial intelligence, robotics, and the Internet of Things are used to improve the productivity, efficiency and sustainability of manufacturing processes [1,2]. This transformation is underpinned by the enhanced collection and use of data, and therefore sensors are one of the most important technologies in Industry 4.0 [3]. Although sensors exist for basic measurements such as temperature and pressure, there is a need for more advanced techniques that can monitor materials and processes. Mixing is one of the most common manufacturing processes. It is not only used for combining materials, but also for increasing heat and mass transfer, providing aeration, and suspending solids. Correct active ingredient dosing in the pharmaceutical industry is critical for patient safety and treatment effectiveness and effective mixing is essential to achieve this. In food manufacturing, mixing provides uniform heating and modifies material structure. In material manufacturing such as the polymer, cement, and rubber industries, final product qualities are determined by the level of homogeneity [4]. Sensors that provide automatic, real-time data acquisition capabilities are required to monitor critical processes such as mixing. These sensors are termed in- or on-line, where in-line methods directly measure the process material with no sample removal, and on-line methods automatically take samples to be analysed without stopping the process [5]. Sensors able to characterise whether a mixture is non-mixed or fully mixed offer benefits of reducing off-specification products, early identification of process upset conditions, and reduced resource consumption from overmixing. Furthermore, techniques able to predict the required time remaining until mixing completion would improve batch scheduling and therefore process productivity.

There are numerous in-line and on-line techniques available to monitor industrial mixing processes, with the major categories of techniques being point property measurements, tomographic (e.g., electrical resistance tomography), and spectroscopic (e.g., Near Infrared Spectroscopy (NIRS)). Discussion of the aptitude of each technique to different mixing applications is provided in [4]. Active acoustic techniques introduce sound waves into a material or system by converting electrical signal pulses into pressure waves using piezoelectric transducers. Either a single transducer sends and receives the sound wave after reflection from an interface (pulse–echo mode) or a second transducer receives the sound wave after it has been transmitted through the material (pitch catch mode) [6]. Low power, high frequency sound waves in the ultrasonic frequency range are used for material characterisation, and do not affect the structure of the material [6]. Typical ultrasonic parameters measured to characterise a system include the speed of sound, sound wave attenuation, and the material’s acoustic impedance. The speed of sound through the material is dependent on its density and compressibility, and is calculated by measuring the time of flight of the sound wave. The attenuation of the sound wave can be measured as a decrease in the signal amplitude, and is caused by sound wave scattering, reflection, or energy dissipation. The acoustic impedance is dependent on the speed of sound and density of the material, and the proportion of reflected sound wave from a material boundary is dependent on the magnitude of the acoustic impedance mismatch between the neighbouring materials [7]. Ultrasound sensors are low-cost, real-time, in-line, and capable of operating in opaque systems. However, the large changes in acoustic impedance when transmitting from liquid or solid to gas causes strong reflection of the sound wave, making transmission difficult in the presence of gas bubbles. Furthermore, the speed of sound in a material is strongly dependent on temperature [7]. Ultrasound has found application for material characterisation in industries such as food, chemicals, pharmaceuticals, and biotechnology [6,7,8,9].

Several studies have used ultrasonic measurements to monitor mixing. However, many of these require transmission of the sound wave through the mixture in order to measure the speed of sound or attenuation. Stolojanu and Prakash [10] used two invasive transducers in the pitch–catch mode to characterise glass bead suspensions up to concentrations of 45 wt % in a laboratory scale mixing system. The ultrasonic velocity, attenuation, and peak frequency shift were used to determine particle concentration and size. Both Ribeiro et al. [11] and Yucel and Coupland [12] used two non-invasive transducers in the pitch–catch mode to characterise laboratory scale systems. However, transmission-based measurements are unable to be used for most mixing systems at the industrial scale. Firstly, the increased distance that the sound wave must travel increases the attenuation of the signal. Secondly, industrial mixtures are typically more complex than simple model systems tested at laboratory scale. The number of materials being mixed in industrial mixers creates an increased number of heterogeneities causing scattering and reflection of the sound, or the presence of gas bubbles cause strong reflection of the sound wave. These also contribute to greater attenuation of the signal and transmission becomes more difficult without high power, high cost transducers.

Bamberger and Greenwood [13] mounted pitch–catch mode transducer pairs to a probe to monitor solids suspension in an industrial slurry mixing tank. However, this technique was invasive and the attenuation correlation with solids concentration was only possible over the short sound wave propagation distance. Sun et al. [14] monitored the dispersion homogeneity of calcium carbonate in polypropylene during extrusion. Two transducers in the pitch–catch mode measured the ultrasound attenuation. Again, this was invasive and transmission was only possible due to the short sound wave propagation distance. Fox et al. [15] and Salazar et al. [16] used the invasive pulse–echo mode ultrasound probes to monitor air incorporation into aerated batters during mixing. Due to the strong reflectance of sound waves caused by gas bubbles, transmission was not possible. The acoustic impedance of the probe-batter interface was measured to determine the optimal mixing time. Hunter et al. [17] and Bux et al. [18] used intrusive pulse–echo transducers to monitor particle suspension. Acoustic backscatter techniques were used to measure speed of sound and attenuation, where the reflected sound wave from the particles was measured opposed to transmission through the suspension. Invasive techniques suffer from problems such as probe fouling, probe breakage, and difficulty in installation, thereby limiting their appeal in industrial settings. Ultrasound is applicable for non-invasive measurement by transmitting the sound wave through the wall of the vessel. Therefore, this current work uses a non-invasive, pulse–echo ultrasound technique to monitor mixing, which requires no sound wave transmission through the mixture being characterised. The only examples of non-invasive, no-transmission ultrasonic sensors for mixing processes are those used to monitor particle suspension. Buurman et al. [19] used non-invasive ultrasonic Doppler velocimetry to detect whether particles were suspended at the bottom of an opaque mixing vessel to monitor particle suspension. Zhan et al. [20] used a non-invasive pulse–echo transducer attached to the base of the vessel to monitor particle suspension by measuring the acoustic impedance of the base-suspension interface.

In this study, two laboratory-scale mixing systems are monitored: honey-water mixing and flour-water batter mixing. These two model systems were selected to show the application of ultrasonic sensors to monitor different mixing processes. As honey is completely miscible in water, this system is representative of the development of homogeneity in liquid–liquid blending. Flour-water batter was used in this study to monitor structural changes as the gluten proteins in the flour become hydrated and aligned into a network, as opposed to air incorporation as investigated in Fox et al. [15] and Salazar et al. [16]. Therefore, this flour-water batter system is similar to dough mixing, only with higher water content. This system was chosen as during dough mixing at atmospheric pressure, the dough pulls away from the mixer sides and is therefore not measurable using low-power ultrasound due to the created air gap. However, industrial dough mixing is typically performed at reduced pressure or vacuum pressure, where the dough will be in contact with the mixer sides. Furthermore, batter mixing has been shown to follow the same physical and chemical changes as dough during mixing, and is therefore representative of industrial dough mixing [21].

For in-line industrial process monitoring, suitable signal processing and interpretation is required for automatic process diagnosis. Supervised Machine Learning (ML) maps input data to output classes (classification) or values (regression) during training so that it may then be used to predict outputs from new input data. The advantage of ML is the ability to fit functions to input–output relationships without the need to define the often complex underlying physical models. The success of ML models is dependent on the input feature variables used to make predictions. A received ultrasonic waveform consists of an amplitude at each time period sample. From this waveform, useful features are typically manually engineered, e.g., selecting the maximum waveform amplitude, or monitoring the speed of the sound wave. This approach of using manually engineered features is termed shallow ML. Ultrasonic measurements have been combined with shallow ML algorithms such as Artificial Neural Networks (ANNs) [22,23,24,25,26,27,28,29] and Support Vector Machines (SVMs) [23,25,30,31], using waveform features from the time domain [23,25,27,31,32] and frequency domain [24,27,31,32] after analyses such as wavelet transforms [22,24]. These have been used for applications such as predicting sugar concentration during fermentation [33], measuring particle concentration in multicomponent suspensions [34], and classification of heat exchanger fouling in the dairy industry [23,25]. There are no examples of using ultrasonic measurements and ML to follow a mixing process; however, El-Hagrasy et al. [35] used the Soft Independent Modelling of Class Analogies (SIMCA) and Principal Component Modified Bootstrap Error-adjusted Single-sample Technique (PC-MBEST) algorithms to analyse NIRS spectra during pharmaceutical solids blending. Typically, shallow ML requires some expertise of the sensor signal to engineer useful features from the raw data. In contrast, Convolutional Neural Networks (CNNs) utilise representation learning, which requires no manual feature engineering by transforming the raw data into higher, more abstract levels to automatically extract features [36]. CNNs use convolutional filters to measure the spatial relationship data values and have found application in image recognition tasks [37,38]. CNNs have also been used to improve ML prediction from ultrasonic signals in both the time [26] and frequency domain after the wavelet transform [39]. The focus of this study is to compare different feature engineering methods and ML algorithms to classify the mixture state and predict the time remaining until mixing completion for two model mixing systems. ANNs, SVMs, and Long Short-Term Memory (LSTM) neural network shallow ML algorithms are compared with CNNs. The wavelet transform will also be investigated to provide the frequency content of the waveforms as inputs to the ML models. The sensors used in this current work only characterise material close to the vessel wall and therefore the potential for non-representative readings must be investigated. This is achieved by comparing the results from multiple low-cost sensors distributed around the vessel along with data fusion between the sensors. Multisensor data fusion is the combination of measurements from multiple sensors to produce improved analysis over that which could be achieved by using the data from each sensor independently.

## 2. Materials and Methods 

### 2.1. Experimental

#### 2.1.1. Ultrasound

**Figure 1:**
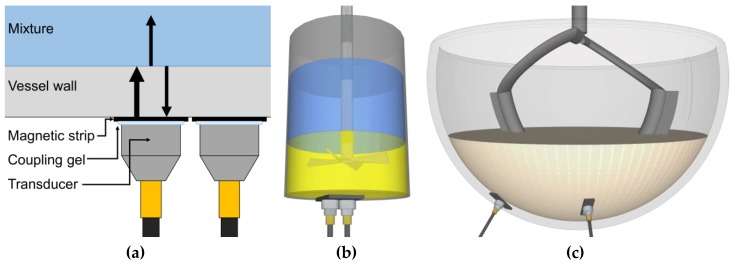
(**a**) At each boundary between two materials, part of the ultrasound wave is reflected. The proportion of the sound wave energy reflected is dependent on the acoustic impedance mismatch between the materials. The ultrasound wave of interest in this study is that reflecting back from the vessel–mixture interface to the transducer. (**b**) Diagram showing the position of the two ultrasound sensors on the vessel base to monitor honey-water blending. (**c**) Diagram showing the position of the two ultrasound sensors on the side of the mixing bowl to monitor the flour-water batter mixing.

Two magnetic transducers of 1 cm^2^ active element surface area with a 5 MHz resonance (M1057, Olympus, -6 DB bandwidth—116.43%) were externally mounted to the mixing vessels. The transducers were attached to adhesive magnetic strips on the outside of the vessels and coupling gel (Proceq ultrasound couplant) was applied between the sensor and strip (Figure 1a). The transducers were used in the pulse–echo mode to both transmit and receive the ultrasonic signal. The ultrasound wave is transmitted through the coupling gel, magnetic strip, adhesive, vessel wall, and mixture. At each interface between different materials, a part of the sound wave continues through to the second material and a part of it is reflected. The proportion of the sound wave reflected at the interface is dependent on the acoustic impedance mismatch between the two materials (Equation (1)) [40]. The ultrasound wave of interest is that reflected from the vessel–mixture interface. The acoustic impedance is a product of the material density and speed of sound (Equation (2)) [40]. Therefore, the acoustic impedance mismatch between the mixture and vessel materials is the parameter being measured; this is depicted by the wave returning from the vessel–mixture interface to the transducer in Figure 1a. During the mixing processes, the composition of the mixture in contact with the vessel wall at the sensor measurement area will change. This will change the acoustic impedance mismatch between the vessel wall and mixture, and therefore this technique is suitable to monitor the mixing process. The portion of the wave that continues through the mixture is either dissipated due to attenuation or reflects from additional boundaries present in the mixture. However, only reflections from boundaries approximately perpendicular to the direction of wave propagation will be received by the transducer. Due to the thin thickness of the vessel wall, the ultrasonic technique is operating in the near field. The near field is the region closest to the transducer element and has a complex structure due to constructive and destructive interference of multiple waves generated by the transducer surface. Although performing measurements in the near field is challenging, it is possible as highlighted by the work of Escrig et al. 2019 [8].

(1) $$R=\frac{A_{r}}{A_{i}}=\frac{Z_{1}-Z_{2}}{Z_{1}+Z_{2}}$$

(2) $$Z_{i}=C_{i}\rho_{i},$$

where *R* is the reflection coefficient, *A_r_* the amplitude of the reflected wave, *A_i_* the amplitude of the incident wave, *Z*_1_ and *Z*_2_ the acoustic impedance of the material the wave is travelling from (material 1) and the material the wave is travelling into (material 2). *C_i_* is the speed of sound in material *i*, and *ρ_i_* the density of material *i*. These parameter values for the tested materials are provided in Table 1.

#### 2.1.2. Honey-Water Blending

The ultrasonic transducers were mounted on the base of a 250 mL glass vessel. This was because a flat surface was needed to allow full contact of the sensors and the magnetic strip. It was not possible to mount the transducers on the side of the vessel due to its curvature. As the sensors only measure a small area of material properties in a single location, they may be designated as point property measurement techniques. Therefore, the positioning of the sensors is of paramount importance to obtain useful readings; for example, multiple NIR sensors have been used to monitor different mixing dynamics during particulate blending across different locations in a mixer [49,50]. Therefore, one sensor was located at the centre of the vessel base and the other was closer to the vessel sides (Figure 1b), allowing comparison between both sensor positions. Furthermore, sensor fusion could be explored by combining outputs from both sensors to improve ML prediction. An ultrasound box (Lecoeur Electronique) was used to excite the transducers by providing electrical pulses and digitisation of the received signals (2048 bit resolution). A temperature sensor was taped to the base of vessel and connected to a PT-104 Data Logger (Pico Technology) to monitor local temperature. The ultrasound box and temperature data logger were connected to a laptop and bespoke MATLAB software controlled the hardware components and acquired the data. Before beginning the experiments, the change in waveform in the presence of water was used to select the portion of the signal reflecting from the vessel–material interface. The ultrasonic gain was then set to maximise the resolution of this portion of the reflected waveform. The number of samples was then also selected by monitoring the last sample point where a change in waveform could be seen visually using this test. Signals were acquired continuously for 1 s from each probe consecutively. Two ultrasonic waveforms were recorded during each 1 s time period. For the ANNs, SVMs, and LSTMs the signal was averaged over this 1 s interval before applying feature engineering to minimise the effect of signal noise. However, for the CNNs every waveform collected during the 1 s intervals was used in training to maximise the number of images that the network had available for training. The sampling frequency was set to 160 MHz to maximise waveform resolution. A mobile phone camera was used to film each mixing process to be later used to determine the time for mixing completion, defined as the time when the honey had fully dissolved. The location for the last part of the honey to be fully mixed was in the centre of the vessel base. This determination of time for mixing completion is the ground truth data to label the output of each ultrasonic waveform. From this labelled data, the ML models can be trained and tested to predict the mixture state (non-mixed or mixed) or predict the time remaining until mixing completion. Pure clear honey (Wm Morrison Supermarkets plc) and tap water were loaded into the vessel at the start of each mixing process. Two different volumes of honey were used for the experiments: 20 and 30 mL. A constant volume of 200 mL tap water was used throughout. An overhead stirrer with a cross-blade impeller was used to stir the mixture. The impeller speed was also set to values of either 200 or 250 rpm. These four parameter permutations were repeated three times across one day while varying the laboratory thermostat set point to produce a temperature variation from 19.3 to 22.1 °C. This induced variability in process parameters was performed to enable the ML models to generalise.

#### 2.1.3. Flour-Water Batter Mixing 

NIRS has previously been used to monitor the chemical and surface structure changes occurring during dough mixing [51,52,53] and image analysis of the dough surface has been used to determine optimal mixing time [54]. Measuring the power or torque supplied to the impeller is a common method of monitoring dough mixing. Mixing should be stopped at the maximum power input for optimal bread properties [54]. Beyond this point of maximum resistance to extension, the gluten network begins to breakdown. The standard deviation of the power measurement has also been found to peak at the optimal dough consistency [55]. Ultrasound has previously been used to characterise the effects of mixing on dough. The relevant literature is included in the Results section. However, none of these ultrasonic techniques have used an in-line monitoring system similar to that used in this investigation. The power supplied to the motor was monitored using a YouThink plug socket power meter to provide a reference measurement for the mixture’s state. The optimal mixing time was determined by the time of maximum power drawn to the impeller. From this, an output value for each sensor signal can be labelled with the ground truth data to then train and test the ML models. The same transducers as used in the honey-water blending experiments were attached to the outside of a stand-mixer glass mixing bowl (1000 W Kenwood kmix kmx754). Due to the curvature of the vessel, the adhesive was not sufficient to hold the magnetic tape onto the sides. Therefore, the magnetic tape was attached to the vessel using silicone vacuum grease and electrical tape. The transducers were located close to the base of the vessel to reduce the likelihood of an air gap caused by the dough pulling away from the mixing bowl sides when close to the optimal mixing time. As both sensors were located at the same height on the mixing bowl, a comparison between different sensor positions could not be made. This is because the mixing was similar at all radial positions in the vessel for the same height position. Rather, the ability of one sensor versus two sensors to monitor the mixing process could be evaluated. The quantity of strong white flour (Wm Morrison Supermarkets plc) and tap water was varied between 450 and 450 g, 500 and 450 g, and 500 and 400 g, respectively. Each combination was repeated three times producing nine runs. Again, this induced variability between runs allowed the examination of the ability of the ML algorithms to generalise across process parameters. A creaming beater was chosen as the attachment to prevent non-mixed zones and the formation of a fouling layer at the surface of the sensor measurement. The minimum impeller speed was used for 1 min to incorporate all of the flour into the water and was then increased to Speed 2 for the remainder of the process. The environmental temperature varied between 19.4 and 21.3 °C. The signal gain and number of sample points were selected in the same process as described in the honey-water blending section. Again, ultrasound signals were acquired continuously for 1 s from each probe sequentially at a sampling frequency of 160 MHz and treated in the same way as previously described.

### 2.2. Data Analysis

All data analysis and ML algorithms were completed in MATLAB R2019a.

#### 2.2.1. Waveform Preprocessing

**Figure 2:**
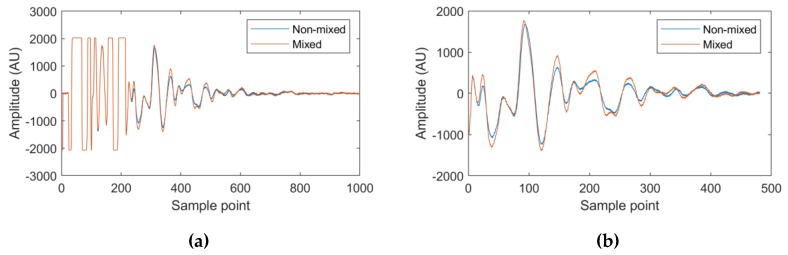
The example waveforms presented are the starting and final waveforms collected from the first run of the honey-water blending experiments. Corresponding to a non-mixed material state and a fully-mixed state, respectively. (**a**) The non-windowed waveform, with the signal saturated from sample points 1 to 210 and containing no further useful information from sample point 700 to the end of the waveform. (**b**) The windowed waveform.

The ultrasonic signal was first windowed to select the useful information from the waveform to use in subsequent calculations and machine learning tasks. Visual inspection of the waveform was used to identify and remove the saturated part of the waveform (sample points 1 to approximately 210 in Figure 2a) corresponding to sound wave reflections prior to the vessel–mixture interface. The part of the waveform displaying no further amplitude change between non-mixed and well-mixed materials (sample point 700 to the end of the waveform in Figure 2a) was also identified and removed from the final waveform (Figure 2b).

#### 2.2.2. Machine Learning Model Development

##### Feature Engineering

A particular focus of this study is to compare feature engineering methods for shallow ML in both the time and frequency domain. The following sections detail the methods used and justify the selection of each.

The waveform Sum Absolute Amplitude (*SAA*) is the summed amplitude magnitude of each sample point in a waveform. It is a measure of the sound wave proportion reflecting back from the vessel–mixture interface and is therefore dependent on changes in mixture acoustic impedance.

(3) $$SAA=\overset{i=SP}{\underset{i=1}{\sum }}\left|A_{i}\right|$$

where *SAA* is the sum absolute amplitude, *SP* is the total number of sample points, and *A_i_* is the amplitude at sample point *i* [34].

The waveform energy is the summed squared amplitudes of every sample point in a waveform. It is therefore a similar measure to the waveform *SAA*; however, it gives greater weight to larger amplitudes. Therefore, two waveforms of the same waveform *SAA* but having different shapes can produce different waveform energies.

(4) $$E=\overset{i=SP}{\underset{i=1}{\sum }}A_{i}{}^{2}$$

where *E* is the waveform energy [34].

Principal component analysis (PCA) was also used throughout the investigation as a feature dimension reduction method. The waveform amplitude at each sample point in the waveform was used as the input variables. Therefore, the obtained principal components (PC) were constructed from parts of the ultrasonic waveform that change at the same time, providing more information than only using the *SAA* or waveform energy. PCA is an unsupervised ML algorithm, meaning only the relationship of input variables to one another are investigated. PCA extracts a new set of orthogonal variables, or PCs, which are a combination of co-linear input variables [56]. When combining the data from both sensors, the sample points in both waveforms were used as input variables. In all cases, the PCs explaining >95% of the variance in the input data set were used as features. If this number happened to be greater than 10 PCs, then only the first 10 were taken as features to prevent overfitting.

The gradients of each feature (e.g., the waveform energy, *SAA*, or PC magnitudes) were investigated for use as additional features that represent previous process time-steps. The difference between consecutive parameter values were calculated after applying a backwards, one-sided moving mean of varying size. A backwards, one-sized gradient means that only past process data is used. The sizes of the moving mean chosen for each ML prediction task is presented in the relevant Results sections.

(5) $$MMV_{i}=\frac{1}{N}\overset{i-N}{\underset{i}{\sum }}V_{i}$$

(6) $$G=MMV_{i}-MMV_{i-1},$$

where *G* is the gradient of a parameter, *MMV* is the moving mean value of a parameter, *N* is the size of backwards, one-sided moving mean, and *V* is the original parameter value [57,58].

While the Fourier transform uses non-decaying sine and cosine waves as transform functions, the wavelet transform uses decaying wavelets (small waves) to analyse the frequency content of a waveform at each location in the time domain [59]. Opposed to the Continuous Wavelet Transform (CWT) analysis, which uses continuous wavelet frequencies as transform functions and therefore produces much redundant information, the Discrete Wavelet Transform (DWT) performs successive decomposition of a waveform by halving the frequency of the orthogonal analytical wavelet, thereby retaining no redundant information after the transform [60]. A key parameter is the choice of the analytical wavelet shape, termed the mother wavelet [61]. The Symlet wavelet was selected owing to it being the least asymmetric, which is most visually similar to the expected waveform composition [62]. The number of vanishing moments and decompositions were investigated and the optimal results for each ML task are reported in the relevant Results sections. After performing the DWT, features for ML model development were engineered in similar ways as described previously. The waveform energy and *SAA* of each decomposition were used as features, or PCA was applied using the waveform amplitude at every sample point in all decompositions as input variables.

##### Training, Validation, and Test Data Set Splits

Throughout this investigation a k-fold testing procedure (where k is the number of runs undertaken for each mixing system; 12 in the case of honey-water blending and 9 for flour-water batter mixing) was carried out by holding one run back for the testing data set. The run held back was alternated between all runs and the models were retrained. The average test set performance was taken as the result for that feature and algorithm combination. The training and validation splits are discussed in each individual algorithm section. The number of data sets obtained from each sensor for each ML method is provided in Table 2 and Table 3.

##### Artificial Neural Networks

Neural networks were investigated for their ability to create new features from input variables, which have a linear relationship with the outputs [36,63]. The constructed ANNs consisted of three layers—an input, hidden, and output layer. The trainlm training function was used for regression networks and the trainscg training function for classification [64]. The training was stopped once the validation loss had increased for 6 consecutive iterations to prevent overfitting. For each neural network, 10 networks were trained and the average performance value was used to account for the effects of random weight initialisation. To further prevent overfitting, the training and validation data set was further broken down into 70% training, 15% validation, and 15% test for an initial hyperparameter optimisation search. A grid search determined the optimal number of neurons in the hidden layer (varied between 1 and 10 in intervals of 1) and regularisation weight (varied between 0.1 and 0.5 in intervals of 0.1) by monitoring this new test set error. The optimal number of hidden neurons and regularisation weight were used for training the final networks. These used 80% of the original training and validation set as the training set and 20% of the original training and validation set as the validation set. The previously discussed k-fold testing procedure was then used to evaluate the final networks.

##### Support Vector Machines

SVMs were chosen for investigation owing to their ability to handle high dimensional feature spaces and thus use kernels functions for non-linear input–output fitting [65]. Bayesian optimisation for 60 evaluations was used to select the box constraint value, kernel scale, kernel function, polynomial order, and whether the inputs were standardised. The acquisition function was chosen to be the expected improvement [66]. The training and validation data set underwent 5-fold validation and was repartitioned after every evaluation to improve generalization performance.

##### Convolutional Neural Networks

The CNNs consisted of 2 convolutional layers [26,39]. The first was either a 2D or 3D convolutional layer containing 8 5 × 5 pixel filters for each sensor input image depending on whether one or two sensor signals were being used as inputs. The second convolutional layer was a 2D convolutional layer containing 16 5 × 5 pixel filters. Padding was applied to keep the input matrices the same size. Batch normalisation was applied after each convolutional layer to aid training, increase the learning rate, and to provide some regularisation. By normalising each mini batch for every layer in the network, each layer does not need to continuously adapt to changing input distributions [67]. Batch normalisation was followed by the ReLu non-linearity function and 2 × 2 pixel max pooling. The training function used was the “adam” function, the initial learning rate was selected as 0.01 with a drop factor of 0.33 after 4 epochs. Training was carried out for 8 epochs, with a mini batch size of 256. The training data was shuffled after every epoch to improve network generalisation. No validation data set was used to maximise the number of datasets that the network fits to. Therefore, a dropout layer was added before the fully connected layer to further prevent overfitting, with the dropout factor varied between 0, 0.1, 0.3, and 0.5. A dropout layer randomly forgets network nodes during training based on the probability specified by the dropout factor. The effect of this is to ensure all nodes contribute to the prediction and improve model generalisation.

##### Time Domain Input CNNs

**Figure 3:**
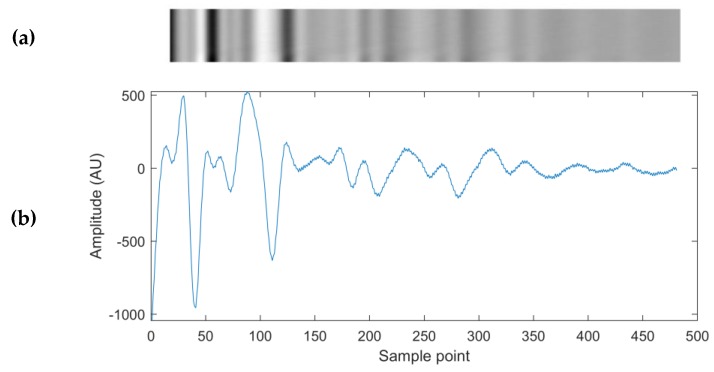
(**a**) A grey-scale representation of the input matrix to the time domain input Convolutional Neural Networks (CNNs), constructed of 25 ultrasound waveforms similar to the waveform depicted below it in Figure 3b. The bright pixels correspond to the maximum amplitude values of the 25 waveforms, and the dark pixels correspond to the minimum amplitude values. (**b**) An example of an ultrasonic waveform used in each row of the time domain input matrix.

The waveform amplitudes at every sample point were used for time domain inputs into CNNs. Input matrices for the CNN were created by stacking 25 windowed signal amplitudes (approximately 10 s of acquired ultrasonic signals) at each time domain sample point on one another, with the current time ground truth being equal to that of the last (bottom) signal (Figure 3). 

##### Frequency-Time Domain Input CNNs

**Figure 4:**
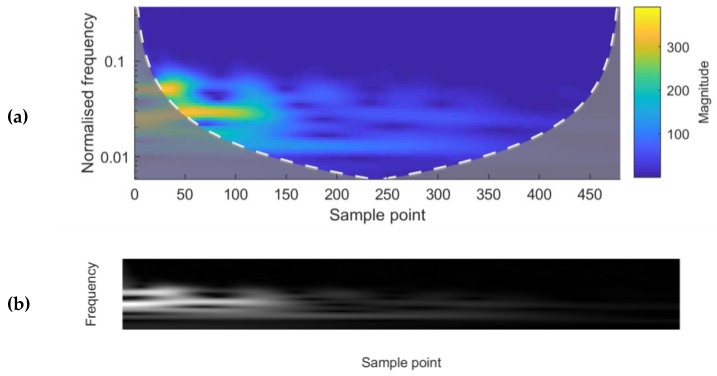
(**a**) A frequency-time domain magnitude scalogram after the Continuous Wavelet Transform (CWT) of a single waveform. The sample points correspond to the sample points of the original waveform. (**b**) A grey-scale image representation of the matrix used as an input to the CNN. It contains the absolute values after the CWT of the same waveform as used for Figure 4a. The bright pixels correspond to the maximum frequency amplitudes, and the dark pixels to the minimum.

The absolute values of the frequency-time domain map after the Continuous Wavelet Transform (CWT) were used as an alternative input to the CNNs. An example of a single waveform after undergoing the CWT is presented in Figure 4. The Morlet wavelet was selected as the mother wavelet owing to the expected symmetry of returning sound waves.

##### Long Short-Term Memory Neural Networks

As this research was interested in monitoring time-evolving processes, LSTMs were an obvious choice for investigation due to their ability to store representations of all previous time-steps in a sequence. LSTM networks are a development of Recurrent Neural Networks (RNN) to overcome problems of exploding and vanishing error gradients. While RNNs use feedback connections to use the output from the previous time-step as an input for the current time-step, LSTMs have gate units to update the internal network state [68]. No validation set was specified to maximise the training data set size for the LSTM networks. The inputs were standardised to give a mean of zero and a standard deviation of 1. The mini-batch size was selected to be 2 runs and the sequence length of each run was sorted to minimise the amount of padding applied. The training was carried out for 60 epochs using the “adam” optimisation algorithm, an initial learning rate of 0.01, and a gradient threshold of 1 to prevent problems of exploding gradients. In the LSTM layer, 50 hidden units were used and 50 neurons in the fully connected layer. These relatively few hidden units and neurons were selected along with a 0.5 probability dropout layer to prevent overfitting and improve algorithm generalisation performance.

## 3. Results

### 3.1. Honey-Water Blending

**Figure 5:**
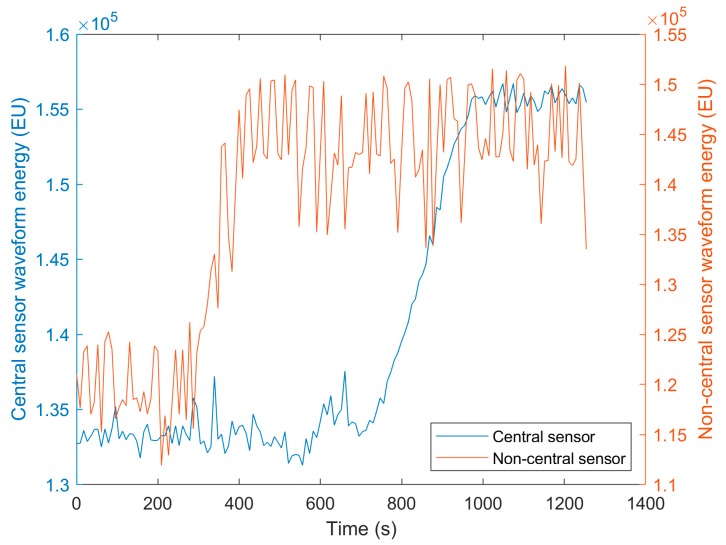
The waveform energy profile during the honey-water blending process for the central and non-central sensors.

**Figure 6:**
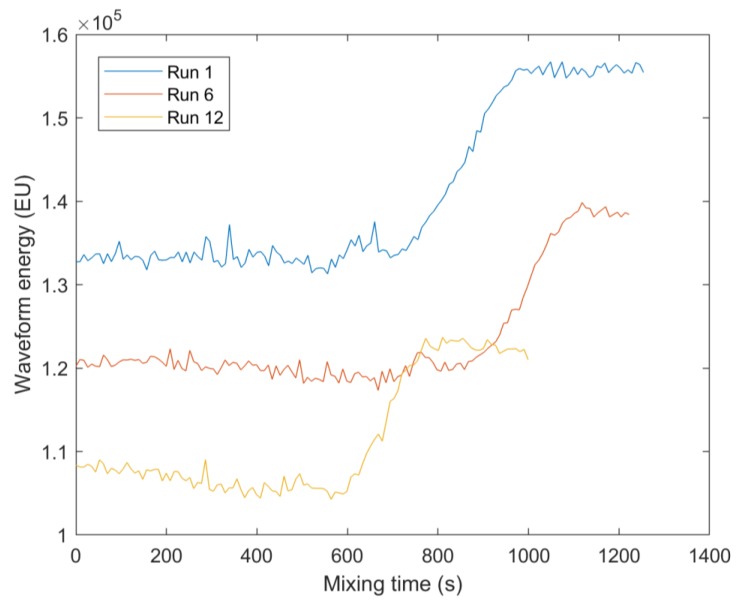
Waveform energy profiles for Run 1 (20 mL honey, 200 rpm impeller speed, 19.4 °C average temperature), Run 6 (30 mL honey, 200 rpm impeller speed, 20.1 °C average temperature), and Run 12 (30 mL honey, 250 rpm impeller speed, 20.7 °C average temperature). Only three runs are presented to aid visibility of the parameter profiles. This figure displays the variation in waveform energy levels in the data, highlighting the need of Machine Learning (ML) methods to monitor the mixing process.

Figure 5 displays the waveform energy profiles of both sensors during Run 1. The waveform energy was lowest at the beginning of the process as honey has a closer acoustic impedance to glass in comparison to water (Table 1, Equation (2)). Therefore, a greater proportion of the sound wave energy transferred into the honey. The waveform energy of the non-central sensor then increases as honey is removed and the water fraction increases at the sensor measurement area. This is because the action of the impeller creates more mixing at the sides of the vessels, rather than directly below the impeller in the centre of the vessel. Finally a plateau is reached when honey has been completely removed from the non-central sensor measurement area. The waveform energy of the central sensor then increases to a plateau as the honey concentration at the measurement area decreases. The central sensor has less fluctuations in waveform energy as in the centre of the vessel base the glass was more flat, providing a vessel–mixture interface perpendicular to the direction of sound wave travel and therefore less variability in the proportion of the sound wave returning to the transducer. The waveform energy profiles for the central sensor during Run 1 and Run 12 are presented in Figure 6. Only these two runs are displayed to aid visibility of the parameter profiles. The differing waveform energy magnitudes are due to Run 1 being performed at an average temperature of 19.4 °C, Run 6 at 20.1 °C, and Run 12 at 20.7 °C. This causes different changes to the speed of sound in each material that the sound wave travels through, thus producing large changes in waveform energy in the reflected sound wave of interest. This highlights the need of using ML techniques to monitor the mixing process, as it can be seen that only using the waveform energy to monitor the mixing process would be insufficient. It can also be seen that the waveform energy during Run 12 continues to decline after peaking, indicating that monitoring the gradient of the waveform energy would also be insufficient for detecting the end of mixing.

### 3.2. Flour-Water Batter Mixing 

**Figure 7:**
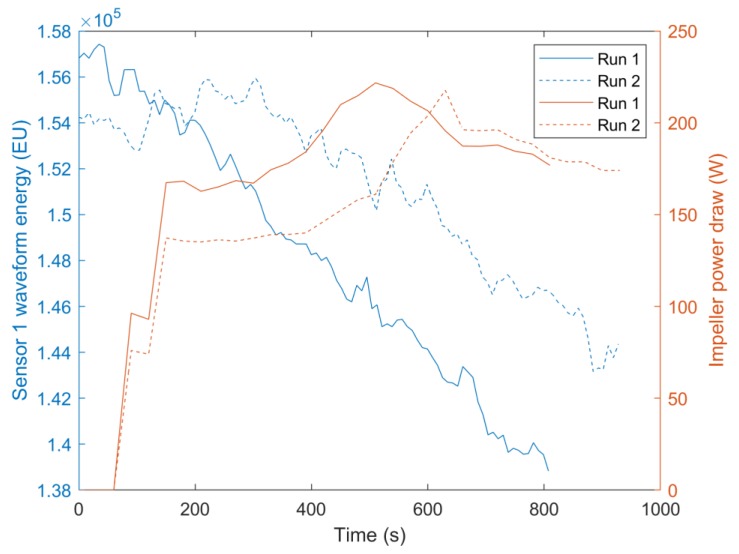
The waveform energy profiles of Sensor 1 and impeller power draw during two flour-water mixing processes containing different volumes of water. Run 1 consisted of 500 g flour and 400 g water and Run 2 500 g flour and 450 g water. Only the waveform energy profiles of Run 1 and Run 2 are provided to aid visibility of the parameter profiles.

In the batter mixing experiments both sensors were located at the same height on the mixing bowl but at different radial positions. The mixing dynamics were similar for all radial positions owing to the mixing bowl being circular and the impeller located in the centre. For this reason, both sensors displayed similar waveform energy profiles for all mixing runs as similar mixtures were present at each sensor measurement area throughout the mixing processes. Therefore, only information from Sensor 1 is presented in Figure 7. Only the waveform energy profiles and impeller power measurements Run 1 and Run 2 are presented in Figure 7 to aid visibility of the parameter profiles. Run 1 consisted of 500 g flour and 400 g water and Run 2 500 g flour and 450 g water. The increased water content of Run 2 delayed the gluten development process and therefore the time of maximum impeller power draw [21]. At the beginning of the process, water or flour may be present at the sensor’s measurement area. If water was first present at the measurement area, the waveform energy initially increases as seen in Run 2. This is because water has a closer acoustic impedance to glass than a poorly mixed flour-water mixture would. The poorly mixed flour-water mixture has a high void fraction, which contains air and therefore produces a low average acoustic impedance over the sensor measurement area. In Run 1, flour was first present at the measurement area so the waveform energy initially decreases. This is because water is mixing into the flour, replacing the air between the flour particles. Ross et al. [44] found a peak in sound wave velocity in bubble free dough at optimal mixing time due to the aligned and fully hydrated glutenin polymers, whereas Létang et al. [45] found the speed of sound in high water content dough (>56% total weight) increases to a plateau at optimal mixing time. Ross et al. [44] found that despite the increasing alignment of the glutenin polymers, there were no significant density changes in bubble free dough during mixing. However, dough is a cellular structure of air incorporated into a viscoelastic matrix during mixing [45,69,70]. Therefore, dough density progressively decreases with mixing time up until the optimum mixing time. Past optimal mixing it begins to increase again as the gluten matrix is broken down by shearing [71]. The decrease in waveform energy (increasing mixture acoustic impedance) up to optimal mixing time marked by the peak in the impeller power draw suggests that the increase in the speed of sound through the dough had a larger effect than this decreasing density (Equation (2)). It should be noted that although increased air entrainment reduces the speed of sound through the dough for lower frequencies [70], at the higher frequencies used here (5 MHz) the ultrasonic velocity approaches the velocity in bubble-free dough, i.e., it travels only through the viscoeleastic matrix [46]. Past the point of optimal mixing, the viscoelastic matrix begins to break down and the water binding capacity of the gluten declines, increasing the level of free water [72]. Although Ross et al. [44] show that this results in a decreasing sound velocity through a regular water content dough matrix, Létang et al. [45] found no change in speed of sound in highly hydrated doughs, suggesting that this increase in free water would not affect this overly hydrated batter. However, the results show a further increase in acoustic impedance, suggesting an increase in batter density past optimal mixing. This is due to the shearing action of the mixer breaking down the polymer matrix and therefore destroying the cellular structure of air pockets, thus increasing the batter density. Again, it can be seen that monitoring the magnitude of the waveform energy would not be sufficient to detect the optimal mixing time, further highlighting the need for ML techniques.

### 3.3. Machine Learning Technique Comparison

#### 3.3.1. Honey-Water Blending Classification

Initial investigations monitored the prediction accuracy of ANNs (as these required the least time to train) to determine the optimal moving mean gradient length, number of vanishing moments of the DWT mother wavelet, and number of DWT decompositions. The optimal size of the moving mean gradient was found to be 25 previous waveforms for the non-central sensor (approximately 25s) and 10 for the central sensor (approximately 10s). The optimal DWT mother wavelet was determined to be Symlets 6 wavelet. This finding was carried forward for all remaining ML tasks. The optimal number of DWT decompositions was found to be 3 for both sensors.

The highest classification accuracy to predict whether the system was mixed was 96.3% and attained using the central sensor and an LSTM with the waveform energy, *SAA*, and gradients as features (Table 4). This superior classification accuracy demonstrates the efficacy of combining ultrasonic sensors with ML techniques. Performing data fusion between both sensors did not provide any benefit over results from using only the central sensor, sometimes even producing lower classification accuracy due to overfitting. This is because the time for mixing completion was defined as the moment the last remaining honey was dissolved. Owing to the motion of the impeller, the location of this event was the centre of the vessel base, where the central sensor was located. Therefore, the highest classification accuracy using the non-central sensor alone was only 89.8%. Although performing the DWT and PCA aided the performance of the ANNs and SVMs, this led to the LSTM neural networks beginning to overfit. Due to their ability to store representations of all previous time-steps, the LSTM neural networks were able to produce the highest classification accuracy using only the waveform energy, *SAA*, and their gradients. ANNs produced their highest classification accuracy when using feature gradients as additional features. However, despite using approximately 10 s of previous time-step waveforms, the time domain input CNNs produced lower classification accuracies than both LSTMs and ANNs (Table 5). This suggests that by using the amplitude at every sample point in the waveform, the time domain input CNNs began to overfit. 

#### 3.3.2. Honey-Water Blending Regression

The optimal moving mean gradient length was found to be 25 previous waveforms for both sensors, and the optimal number of DWT decompositions was found to be three for both sensors.

**Figure 8:**
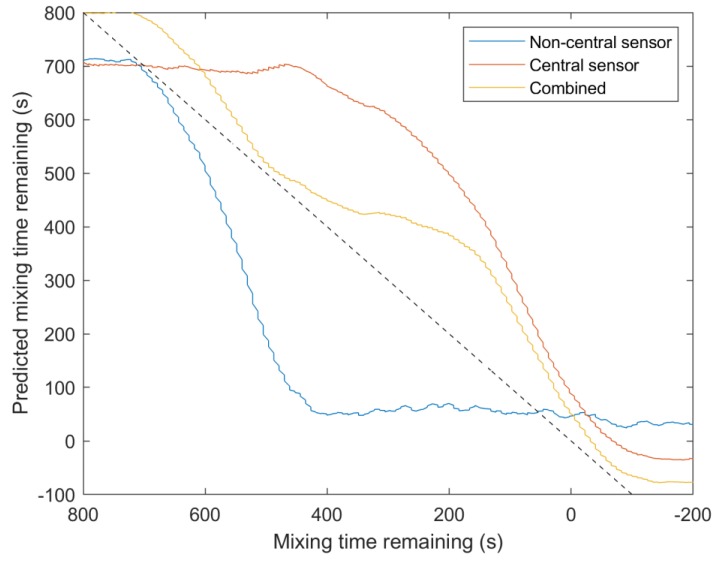
A comparison between regression predictions during honey-water blending for the central sensor, non-central sensor, and combining the outputs from both sensors. A time domain input CNN was used as the learning algorithm. R^2^ values: 0.825 non-central sensor, 0.936 central sensor, and 0.975 multisensor data fusion.

The highest R^2^ values were achieved by combining the inputs from both sensors. R^2^ values of 0.972, 0.973, and 0.977 could be reached using ANNs, LSTMs, or CNNs, respectively (Table 6 and Table 7). This is because the non-central sensor has better prediction ability nearer the beginning of the process as the honey is removed from this sensor measurement area first, and the central sensor has greater resolution nearer the end of the mixing process as the last remnants of honey are dissolved. This is presented in Figure 8, where the combined prediction is more accurate than either single sensor from approximately 600 s before mixing completion until 200 s afterwards. However, reasonable accuracy can be attained using only a single sensor should only one sensor position be available. ANNs using the DWT decompositions energy and *SAA* was able to generalise well and produced an R^2^ value of 0.960 for the central sensor. Similarly, LSTMs using the same features with the non-central sensor achieved an R^2^ value of 0.963, increasing to 0.965 when incorporating feature gradients. Again, these high prediction accuracies illustrate the effectiveness of combining ultrasonic sensors with ML to monitor mixing. As observed when classifying the mixture state, the ability to use data from previous time-steps was vital for accurate prediction of the mixing time remaining. LSTMs, which store representations of all previous time-steps; ANNs using feature gradients; and time domain input CNNs, which use the previous 10 s of acquired waveforms, all produced the greatest R^2^ values with the true mixing time remaining. Time domain input CNNs, which use the amplitude at every sample point in the waveform, displayed the greatest prediction performance. This indicates that, unlike for classifying the mixture state, some useful waveform information is not represented by the time domain and DWT decomposition energies, SAAs, and PCs. However, the CWT input CNNs performed poorer, because only a single waveform was used for prediction. SVMs performed worst overall, most likely because of overfitting due to their convex optimisation leading to a global minima, as opposed to ANNs, which only converge to local minima. Although global cost minimisation is desirable to fit training and validation data, it may lead to poor prediction ability when the test data process parameters lies outside of the bounds of the training data. As in this investigation each monitored run is individually held back for testing, testing on data lying outside of the process parameter space used in training is unavoidable.

#### 3.3.3. Flour-Water Batter Mixing Classification 

The optimal size of the moving mean gradient was found to be 25 previous waveforms for both sensors. The optimal number of DWT decompositions was 7 for Sensor 1 and 5 for Sensor 2.

Both sensors performed equally well and combining sensor inputs did not provide any advantage (Table 8 and Table 9). This shows that the signal received from each sensor was adequate for determining when the system was fully mixed. ANNs and CWT-input CNNs produced the greatest classification accuracies of up to 91.3% and 92.5%, respectively. However, decomposition of the original time domain waveform using wavelet analysis was required to obtain optimal classification. This suggests there was a change in the waveforms only noticeable through wavelet analysis that marked the transition between non-mixed and mixed. Furthermore, the incorporation of previous time-steps in the prediction was not necessary for classification accuracy and may have led to the LSTMs and time domain input CNNs performing poorly due to overfitting. The only highly performing algorithm using past time-steps was ANNs with DWT decomposition energy, SAAs, and gradients as features. The lower classification accuracies for predicting the state of the batter mixture compared with the honey-water blending may be caused by limitations in the frequency analysis employed during this investigation. Further decompositions using the DWT may yield more information in the frequency content of the ultrasonic signals, and an incorporation of an LSTM layer into the CWT input CNNs would provide them with the ability to incorporate previous process states into their prediction.

#### 3.3.4. Flour-Water Batter Mixing Regression

The optimal size of the moving mean gradient and number of DWT decompositions was the same as for the classification of the batter mixture. Despite combined sensor outputs producing the highest prediction accuracies, using both sensors was not required to achieve adequate performance (Table 10 and Table 11). LSTMs using time domain waveform energy, SAAs, and feature gradients achieved an R^2^ value of 0.966, and LSTMs using PCA of the DWT decompositions achieved an R^2^ value of 0.968, both using a single sensor. However, time domain input CNNs produced the highest R^2^ values of up to 0.976 using a single sensor. These results show that performing wavelet analysis was not necessary for high prediction accuracy, unlike for batter mixing classification. Instead, the ability to use previous time-step data and the amplitude of every sample point in the waveform as features is required. However, ANNs preformed worse than both LSTMs and time domain input CNNs, suggesting that a more flexible incorporation of previous time-step data was required for regression accuracy at different process stages rather than using fixed feature gradient lengths throughout the process. Again, the single waveform CWT input CNNs did not produce high prediction performance due to not incorporating previous time-step data as features.

## 4. Discussion

Although ML algorithms were able to achieve similar regression accuracy for both the honey-water blending and flour-water batter mixing, the classification accuracy was lower for the flour-water batter mixing. This is because despite the waveform energies of both processes changing by a similar proportion throughout the mixing processes (Figure 5 and Figure 7), the honey-water blending waveform energy profile has a sharper change during the time of mixing completion. The waveform energy increased as the honey was removed from the measurement area of the central sensor, giving greater resolution of this sensor around the end of the mixing process.

Different ML approaches performed best on each prediction task. To classify the honey-water mixture state, predict honey-water mixing time remaining, and predict the flour-water batter mixing time remaining, the use of previous time-steps as features was useful for prediction accuracy. However, the ability of LSTMs to represent all previous time-states in the internal network, and time domain input CNNs ability to use the previous 10 s of acquired waveforms, performed better than the fixed feature gradient lengths used for the ANNs. In contrast, to classify the mixture state of flour-water batter, no previous time-steps were required. Instead, decomposition of the time domain waveform by the wavelet transform was needed to monitor a state change signature in the frequency domain. Time domain input CNNs were the best performing algorithm to predict the mixing time remaining of both the honey-water blending and flour-water batter mixing. This suggests that the ability to use the amplitude at every sample point in the waveform was better equipped to predict the mixing time remaining than using the waveform energy, *SAA*, or PCs. However, the time domain input CNNs began to overfit when classifying the state of the honey-water mixture. Therefore, the ANN and LSTM prediction accuracy may only sometimes be improved by using the amplitude of all sample points in a waveform. The use of only one acquired waveform for prediction hindered the CWT input CNNs ability to predict the mixing time remaining for both systems, and classified the state of the honey-water blending. Therefore, the addition of an LSTM layer would aid the prediction performance of the CNNs by storing representations of previous time-step data. The only ML task that required combining two sensor outputs was predicting the mixing time remaining for the honey-water blending. This is because the different sensor positions gave increased resolution at different stages of the mixing process. SVMs performed the worst for all prediction tasks. This is likely due to overfitting causing low prediction accuracy on test data outside the parameter bounds of the training and validation data. This is because SVM have convex optimisation functions that produce a global minima. In comparison, ANNs only converge to local minima, which may have aided their ability to generalise to test data outside the parameter space of training.

The application of the combined sensor and ML techniques to monitor processes relies on attaining ground truth data to label the outputs of all sensor signals. In industrial settings, product quality evaluations are typically conducted off-line and require considerable time, expense, or manual operations. This can mean ground truth values to produce labelled data are difficult to obtain, and therefore only a small set of labelled data is available for ML model development. In this case, additional techniques must be considered. For example, semisupervised learning can be used to first perform unsupervised learning on the combined set of labelled and unlabelled data to extract features. Supervised learning models using the labelled data can then be used to predict the class or value of the unlabelled data [73]. Subsequently, active learning can be employed to automatically select data, which would be most useful to the model development if labelled rather than employing annotation of random samples [74]. For example, data points close to classification boundaries or those, which expand the model training space. Transfer learning is another technique that can help overcome the limitation of small labelled data sets. It has found particular application for transferring pretrained CNNs for image recognition tasks or for NIR spectroscopy calibration transfer across spectrometers [75,76]. A model trained on another system, for example a laboratory or pilot scale model system, can be used to aid in the prediction of the state of the target system. For example, the optimised signal processing, network weights, or ML hyperparameter values from the first system can be used as initial training values for the target system. Alternatively, the outputs of the previously trained model applied to the target system may be used as inputs to a second model [75].

## 5. Conclusions

This work studied the potential of using an industrially applicable ultrasonic sensing technique combined with ML to monitor the mixing of two model systems. Two ultrasonic sensors were used for data acquisition, and different ML and feature engineering methods were compared. This work has shown the potential of using ultrasonic sensors and ML to predict the time remaining until mixing is complete and when a system is mixed. The superior prediction accuracies of up to 96.3% for honey-water blending and 92.5% for flour-water batter mixing, along with R^2^ values of up to 0.977 for honey-water blending and 0.968 for flour-water batter mixing, highlight the efficacy of combining ultrasonic sensors and ML to monitor mixing processes. 

## Figures and Tables

**Table 1:** The speed of sound and density of the materials used in the honey-water blending experiments. The acoustic impedance is a product of these two values (Equation (2)). The reflection coefficient is the proportion of the sound wave reflected at the glass vessel wall and mixture interface.

Material	Speed of Sound (m/s)	Density (kg/m^3^)	Acoustic Impedance (×10^6^ Pa.s/m^3^)	Reflection Coefficient
Water	1493 [41]	998	1.49	0.79
Honey	2125 [42]	1420 [43]	3.02	0.61
Well-mixed flour-water mixture (50 wt % water)	2000 [44,45]	1230 [46]	2.46	0.67
Glass, Pyrex	5640 [47]	2210 [48]	12.46	-

**Table 2:** The number of data sets recorded for the honey-water blending process.

Run	The Number of Data Sets from Each Sensor		
	Shallow Learning	Time Domain Input CNNs	CWT Input CNNs
1	146	243	292
2	131	213	262
3	108	167	216
4	166	283	332
5	139	227	276
6	141	233	282
7	109	169	218
8	122	195	244
9	108	165	214
10	102	155	204
11	114	177	226
12	115	179	228
Total	1501	2406	2994

**Table 3:** The number of data sets recorded for the flour-water batter mixing process.

Run	The Number of Data Sets from Each Sensor		
	Shallow Learning	Time Domain Input CNNs	CWT Input CNNs
1	93	137	186
2	90	131	180
3	107	165	214
4	105	159	208
5	102	153	202
6	102	155	204
7	123	197	246
8	129	207	256
9	154	259	308
Total	1005	1563	2004

**Table 4:** Classification accuracies of shallow machine learning algorithms to predict whether the honey-water blending mixture was mixed or non-mixed. E—Energy, SAA—Sum Absolute Amplitude, G—Gradients of Features, PCs—Principle Components, DWT—Discrete Wavelet Transform.

	ANN (% Correct)			SVM (% Correct)			LSTM (% Correct)		
Features	Non-central	Central	Combined	Non-central	Central	Combined	Non-central	Central	Combined
E, SAA	65.5	85.0	83.4	80.2	80.1	86.0	89.5	93.0	88.0
E, SAA, G	76.1	91.1	92.5	77.2	91.1	89.1	89.8	96.3	95.4
PCs	76.2	90.7	83.1	75.1	81.8	80.4	82.9	86.1	91.5
PCs, G	79.3	93.0	92.7	71.8	83.7	86.7	86.2	89.4	93.7
DWT, E, SAA	79.2	91.7	90.6	72.1	82.3	86.4	77.8	95.1	92.9
DWT, E, SAA, G	80.9	92.4	94.6	82.9	92.1	91.9	80.8	94.5	90.8
DWT, PCs	71.9	88.7	90.0	76.2	82.7	80.1	82.5	84.5	89.5
DWT, PCs, G	80.5	95.0	93.9	75.0	85.3	91.0	79.7	86.1	90.4

**Table 5:** Classification accuracies of CNN algorithms to predict whether the honey-water blending mixture was mixed or non-mixed.

CNNs	Time Domain Input (% Correct)			CWT Input (% Correct)		
Dropout layer probability	Non-central	Central	Combined	Non-central	Central	Combined
0	71.7	92.2	93.0	73.5	88.6	88.2
0.1	75.3	92.6	93.1	75.4	89.1	87.0
0.3	72.9	92.7	91.3	73.1	88.2	85.4
0.5	76.3	93.1	93.0	74	90.0	85.4

**Table 6:** Regression accuracies of shallow machine learning algorithms to predict the mixing time remaining of the honey-water blending process. E—Energy, SAA—Sum Absolute Amplitude, G—Gradients of Features, PCs—Principle Components, DWT—Discrete Wavelet Transform.

	ANN (R^2^)			SVM (R^2^)			LSTM (R^2^)		
Features	Non-central	Central	Combined	Non-central	Central	Combined	Non-central	Central	Combined
E, SAA	0.751	0.788	0.920	0.276	0.223	0.795	0.852	0.818	0.954
E, SAA, G	0.875	0.806	0.922	0.894	0.663	0.780	0.755	0.853	0.969
PCs	0.810	0.882	0.956	0.622	0.503	0.817	0.705	0.584	0.959
PCs, G	0.910	0.904	0.972	0.771	0.190	0.721	0.939	0.936	0.969
DWT, E, SAA	0.862	0.960	0.949	0.713	0.570	0.907	0.963	0.895	0.957
DWT, E, SAA, G	0.758	0.892	0.948	0.827	0.722	0.899	0.965	0.865	0.959
DWT, PCs	0.786	0.881	0.957	0.552	0.551	0.806	0.857	0.751	0.973
DWT, PCs, G	0.916	0.914	0.972	0.787	0.503	0.854	0.914	0.930	0.972

**Table 7:** Regression accuracies of CNN algorithms to predict the mixing time remaining of the honey-water blending process.

CNNs	Time Domain Input (R^2^)			CWT Input (R^2^)		
Dropout layer probability	Non-central	Central	Combined	Non-central	Central	Combined
0	0.825	0.936	0.975	0.786	0.866	0.950
0.1	0.828	0.943	0.974	0.790	0.869	0.955
0.3	0.827	0.933	0.974	0.793	0.869	0.951
0.5	0.828	0.932	0.977	0.789	0.866	0.949

**Table 8:** Classification accuracies of shallow machine learning algorithms to predict whether the flour-water batter mixture was fully mixed or non-mixed. E—Energy, SAA—Sum Absolute Amplitude, G—Gradients of Features, PCs—Principle Components, DWT—Discrete Wavelet Transform.

	ANN (% Correct)			SVM (% Correct)			LSTM (% Correct)		
Features	Sensor 1	Sensor 2	Combined	Sensor 1	Sensor 2	Combined	Sensor 1	Sensor 2	Combined
E, SAA	78.5	77.3	81.7	69.9	66.2	78.2	76.9	77.9	80.9
E, SAA, G	79.9	80.6	82.8	79.2	84.2	81.4	85.2	89.6	85.5
PCs	86.9	90.4	89.7	75.7	86.7	83.1	76.1	85.3	86.3
PCs, G	85.5	90.3	86.5	74.0	87.3	76.9	74.6	83.0	84.2
DWT, E, SAA	90.7	90.8	90.8	84.3	88.5	87.7	87.0	85.2	89.3
DWT, E, SAA, G	91.1	90.4	90.0	84.7	89.5	82.0	83.7	88.1	88.3
DWT, PCs	91.3	89.6	91.1	80.4	87.9	84.2	77.7	80.5	88.1
DWT, PCs, G	85.0	88.2	88.4	70.4	72.0	80.9	75.8	84.2	83.4

**Table 9:** Classification accuracies of CNN algorithms to predict whether the flour-water batter mixture was fully mixed or non-mixed.

CNNs	Time Domain Waveforms (% Correct)			CWT (% Correct)		
Dropout layer probability	Sensor 1	Sensor 2	Combined	Sensor 1	Sensor 2	Combined
0	82.6	84.4	83.4	91.5	92.5	90.3
0.1	86.6	85.0	78.7	90.3	90.6	92.2
0.3	85.3	82.4	85.8	87.2	91.6	92.2
0.5	85.7	86.0	78.9	88.4	92.3	92.4

**Table 10:** Regression accuracies of shallow machine learning algorithms to predict the mixing time remaining for the flour-water batter mixture. E—Energy, SAA—Sum Absolute Amplitude, G—Gradients of Features, PCs—Principle Components, DWT—Discrete Wavelet Transform.

	ANN (R^2^)			SVM (R^2^)			LSTM (R^2^)		
Features	Sensor 1	Sensor 2	Combined	Sensor 1	Sensor 2	Combined	Sensor 1	Sensor 2	Combined
E, SAA	0.633	0.697	0.868	0.208	0.430	0.658	0.937	0.935	0.936
E, SAA, G	0.576	0.819	0.835	0.712	0.817	0.873	0.912	0.966	0.974
PCs	0.846	0.879	0.855	0.464	0.775	0.810	0.732	0.822	0.848
PCs, G	0.947	0.91	0.946	0.485	0.713	0.629	0.641	0.760	0.754
DWT, E, SAA	0.831	0.872	0.876	0.392	0.781	0.535	0.940	0.950	0.912
DWT, E, SAA, G	0.666	0.766	0.743	0.815	0.917	0.631	0.771	0.953	0.955
DWT, PCs	0.844	0.898	0.824	0.489	0.665	0.485	0.932	0.968	0.958
DWT, PCs, G	0.840	0.930	0.906	0.662	0.774	0.654	0.786	0.911	0.939

**Table 11:** Regression accuracies of CNN algorithms to predict the mixing time remaining for the flour-water batter mixture.

CNNs	Time Domain Waveforms (R^2^)			CWT (R^2^)		
Dropout layer probability	Sensor 1	Sensor 2	Combined	Sensor 1	Sensor 2	Combined
0	0.961	0.976	0.975	0.945	0.920	0.958
0.1	0.962	0.973	0.980	0.944	0.922	0.962
0.3	0.959	0.970	0.982	0.938	0.922	0.958
0.5	0.960	0.975	0.977	0.940	0.915	0.961
